# The peritonsillar abscess and its management – is incision and drainage only a makeshift to the tonsillectomy or a permanent solution?

**DOI:** 10.3389/fmed.2023.1282040

**Published:** 2023-11-29

**Authors:** Manuel Christoph Ketterer, Maren Maier, Valentin Burkhardt, Naglaa Mansour, Andreas Knopf, Christoph Becker

**Affiliations:** Department of Otorhinolaryngology, Medical Center – University of Freiburg, Faculty of Medicine, University of Freiburg, Freiburg, Germany

**Keywords:** Peritonsillar abcess, surgical procedures, incision and drainage, tonsillectomy, complication

## Abstract

**Introduction:**

This study aims to examine the long-term management of peritonsillar abscess and compare needle aspiration, incision with drainage, and tonsillectomy in terms of comorbidities, complication rates, and recurrences in the largest study cohort published to date.

**Methods:**

We conducted a retrospective analysis of patients, both adults and children, who were treated for peritonsillar abscess between 2007 and 2019. Patient charts were analyzed to assess surgical treatment, infection and inflammation rates, risk of bleeding, recurrence rates, duration of illness, and sick certificates. Additionally, patient imaging and blood levels were compared. Postal questionnaires were sent to all patients to evaluate subjective success rates, complications, and long-term benefits of the different treatment regimens. General practitioners and ENT doctors in private practices were contacted to gather missing data on the long-term course of the disease.

**Results:**

A total of 821 patients with peritonsillar abscess were included in this study. Two patients had to be excluded due to incidental pathological findings. Of the remaining 819 patients, 180 were successfully treated with needle aspiration or incision. Among these patients, 37.7% required tonsillectomy during the same inpatient stay. Laboratory parameters such as leukocyte count or C-reactive protein levels were not indicative of the need for tonsillectomy. Furthermore, computed tomography was only necessary in cases of suspected parapharyngeal abscess, not in clear cases of peritonsillar abscess. Among the 641 patients who underwent tonsillectomy, 11.4% experienced postoperative bleeding requiring treatment. Only patients who underwent bilateral tonsillectomy reported recurrent episodes of sore throat and pharyngitis resulting in absence from work. The ipsilateral recurrence rate for peritonsillar abscess after needle aspiration or incision was 2.8%. There were no contralateral recurrences during the observation period.

**Conclusion:**

Due to the lower risk of postoperative bleeding, shorter absence from work, and shorter inpatient stay, incision and drainage are the preferable treatment for peritonsillar abscess. Additionally, patients who underwent bilateral tonsillectomy reported higher rates of work incapacity due to sore throat caused by pharyngitis. No patient met the clear indication for bilateral tonsillectomy due to recurrent acute tonsillitis. The recurrence rate after drainage without tonsillectomy was very low (2.8% ipsilaterally, no recurrence contralaterally).

## Introduction

Peritonsillar abscess (PTA) is the most common deep infection in the field of otorhinolaryngology, with an incidence of 30 cases per 100,000 individuals per year. It is a frequent clinical condition not only for otorhinolaryngologists but also for primary care physicians (1, 2). Complications can arise, leading to airway obstruction, deep neck and chest soft tissue infections, or mediastinitis (1). For a long time, abscess tonsillectomy (TE) was considered the surgical treatment of choice. However, like elective TE, abscess TE carries a high risk of secondary bleeding (3). Additionally, abscess TE poses a higher risk of bacteremia and subsequent spread of bacteria, including the risk of endocarditis, compared to elective TE (4). Needle aspiration (=NA) and incision with drainage (=ID) under local anesthesia are possible therapeutic alternatives, but they may not be suitable for all cases, particularly depending on the abscess localization (5–7). Some authors have reported that intravenous antibiotic therapy alone, without surgical treatment, is sufficient in most cases (8). However, they did not examine the recurrence rate among these patients who later required further surgical therapy.

In recent years, the management of PTA has shifted from direct abscess TE to incision and drainage (=ID), which is increasingly utilized. Since abscess TE is associated with an increased risk of up to 22% of secondary bleeding (9), patients often experience an extended hospital stay in Germany. Postoperative pain is typically higher, and the procedure is usually performed under general anesthesia. Nevertheless, when performed correctly, abscess TE provides definitive drainage and therapy by removing the entire front wall of the abscess, significantly reducing the chances of re-abscess formation. On the other hand, NA or ID of the abscess can be effectively carried out under local anesthesia with less peri- and post-interventional pain. The risk of secondary bleeding is also significantly lower (9). However, there may be an increased risk of re-abscess formation because the anterior wall of the abscess cavity is not removed.

To date, there is a lack of literature with sufficient follow-up time to assess the recurrence rate after NA, ID, or abscess TE (German guideline). Therefore, the aim of this study is to investigate a large patient cohort over a long post-therapeutic period. We evaluated the extent to which NA or ID under local anesthesia can serve as sufficient and permanent therapies for PTA and determined the recurrence rate among these patients who subsequently required abscess TE. Furthermore, we examined the number of patients who underwent contralateral TE during this interval. Additionally, we sought to determine which of these patients met the guideline indication for recurrent tonsillitis and how many of the patients who underwent TE experienced recurrent pharyngitis afterward.

## Methods

### Study cohort

This study presents a retrospective analysis of pediatric and adult patients who were treated at a quaternary university hospital over the past 12 years (2007–2019). The patients were divided into four groups based on their treatment: needle aspiration (NA-group), incision and drainage (ID-group), unilateral abscess TE (UTE-group), or bilateral abscess TE with elective TE on the other side (BTE-group) for unilateral PTA. Our hypothesis was that patients who underwent NA or ID would have a significantly higher risk of PTA recurrence and therefore require abscess TE. Additionally, we assumed that patients who had unilateral abscess TE would be at an increased risk of developing a contralateral PTA. As a secondary aim of this study, we investigated whether patients undergoing TE would experience pharyngitis more frequently after the procedure.

Patients with a clear documentation of retrotonsillar abscess or intratonsillar abscess, and those who did not receive simultaneous antibiotic treatment or ended antibiotic treatment prematurely were excluded from the study. We also excluded patients who underwent elective TE for recurrent acute tonsillitis and patients with tonsil malignancies.

### Data collection and patients’ consent

Patients’ charts were retrospectively evaluated for demographic variables (age, sex, date of inpatient stay), specific characteristics (histopathology, treatment), and follow-up data. We reviewed operation reports, course documentation, doctors’ letters, radiological findings (computed tomography (=CT) and magnetic resonance imaging), laboratory findings, ICD/OPS coding (International Statistical Classification of Diseases and Related Health Problems/Procedure Key), and pathological results. Written consent was obtained from all patients included in the study. They were informed about the study through postal communication and asked for their consent. Questionnaires were provided to the patients in the NA- and ID-groups, as well as the UTE- and BTE-groups, to assess the rates of sore throat, pharyngitis, bleeding, and subsequent treatment following their PTA in the last 12 years. Additionally, we contacted their general practitioners and, if possible, their ENT doctors in private practice to gather information on the rates of sore throat, pharyngitis, tonsillitis, and obtain doctor’s certificates and sick notes related to these conditions.

### Statistical analysis and ethics committee

Statistical analyses were performed using SPSS (SPSS Inc., Chicago, IL), and *p*-values below 0.05 were considered statistically significant. Differences between the groups were analyzed using the Chi-square test and Fisher exact test for categorical variables, and the unpaired Student’s t-test for continuous variables. Normality of value have been verified before further analysis. Time trends were analyzed using a linear regression model. This study was conducted in the Department of Otorhinolaryngology, Medical Center – University of Freiburg, Faculty of Medicine, University of Freiburg, Germany. The study was approved by the Hospital’s Ethics Committee in accordance with the Declaration of Helsinki (10) (Approval number: 154/20; Amendment number: 201653). This retrospective study is registered in the German Clinical Trials Register (DRKS = Deutsches Register für klinische Studien. Number: DRKS00030619).

## Results

### Study cohort, comparison of treatment options, and diagnostic tools

**Figure 1:**
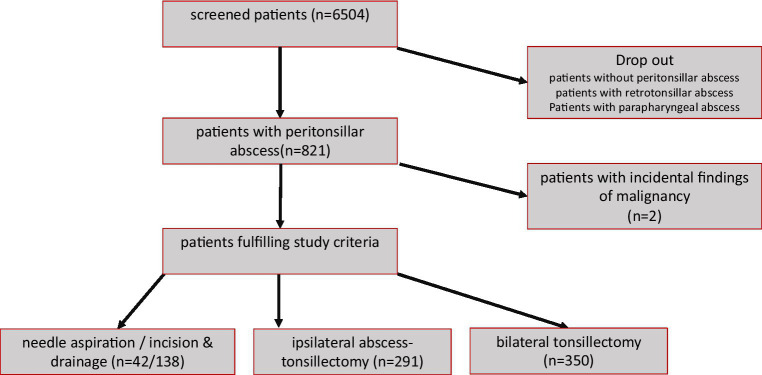
Visualization of study inclusion and screening.

In this retrospective analysis, we included 821 patients out of the initially screened 6,504 patients (please see Figure 1: flow chart). The mean age at PTA treatment was 36.1 +/− 17.7 years (maximum: 93.4 years; minimum: 1.8 years). Considering a mean age of 43.5 years, we believe that long-term benefit and success rates have been adequately evaluated (see Table 1). The questionnaire return rate was 22%. Among the patients, 641 showed histopathologic findings consistent with inflammatory changes. However, two patients had incidental findings and were excluded from further analysis of the study, one with acute myeloid leukemia and one with B-cell lymphoma; remaining 819 patients for further analysis. All included patients were treated with antibiotics, with a preference for cefuroxime and metronidazole. In cases of swelling, prednisolone was administered.

**Table 1:** Distribution table and study cohort in total: (PTA = peritonsillar abscess, SD = standard deviation, CRP = C-reactive protein, NA-group = needle aspiration group, ID-group = incision and drainage group).

Total (*n*)	821			
Time (year)	2007–2019			
	Mean	Minimum	Maximum	SD
Age at study analysis	43.5	8	101	17.6
Age at treatment of the PTA	36.1	2	93	17.8
Leucocytes in total	14.07	0.4	32.7	4.62
CRP in total	94.99	3	478	78.2
Pathology	Fitting: 641		Incidental finding: 2 (AML/lymphoma)	
Rate of return of the questionnaires (*n*)	Total: 181 (22%)			
	NA-/ID-group: *n* = 45 (33%)			
	Tonsillectomy: *n* = 136 (21.2%)			

**Figure 2:**
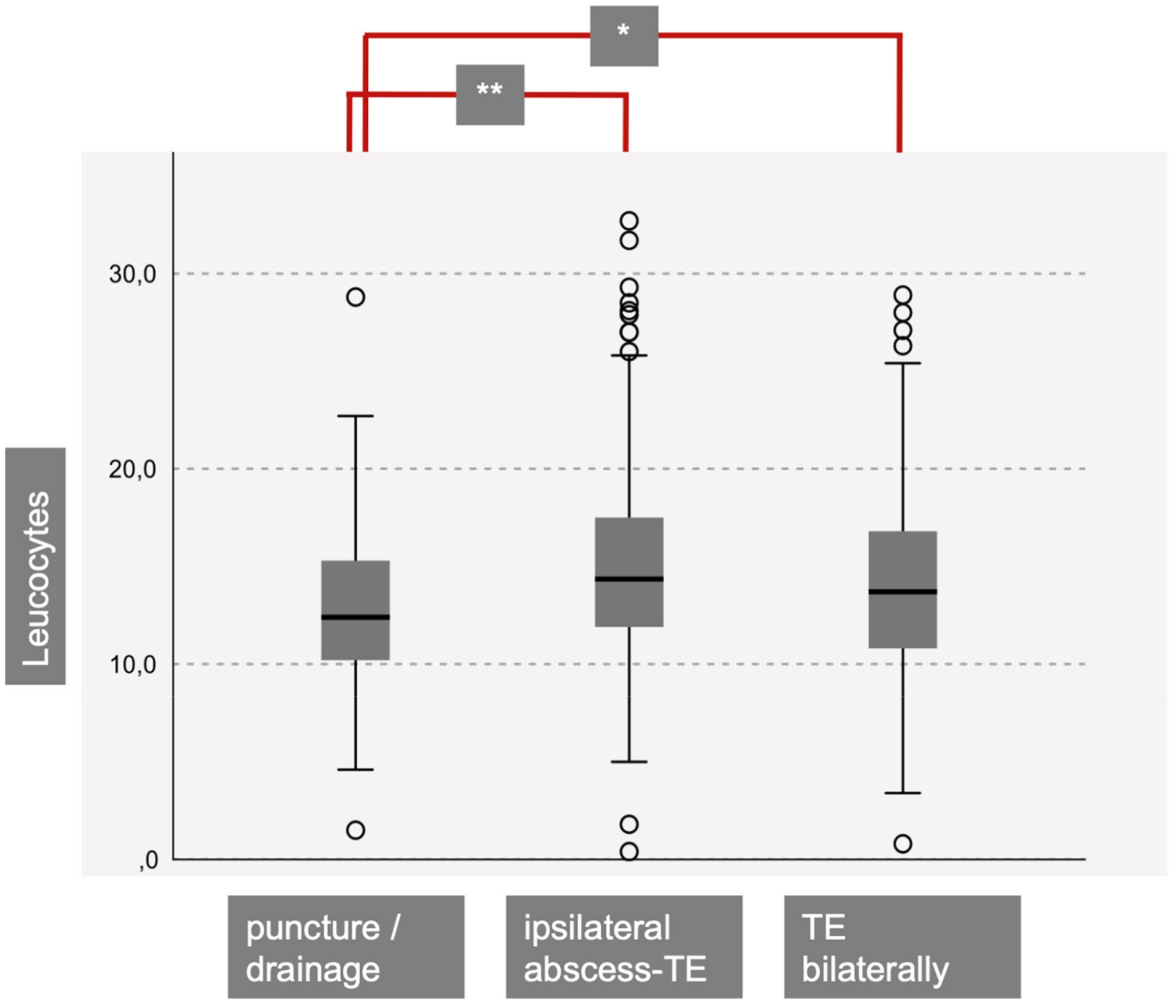
Patients with a successful needle aspiration (puncture) or incision and drainage showed significantly diminished leucocytes at initial presentation compared to patients with ipsilateral abscess-TE (*p* < 0.001) and bilateral TE (*p* = 0.04) (TE = tonsillectomy).

**Figure 3:**
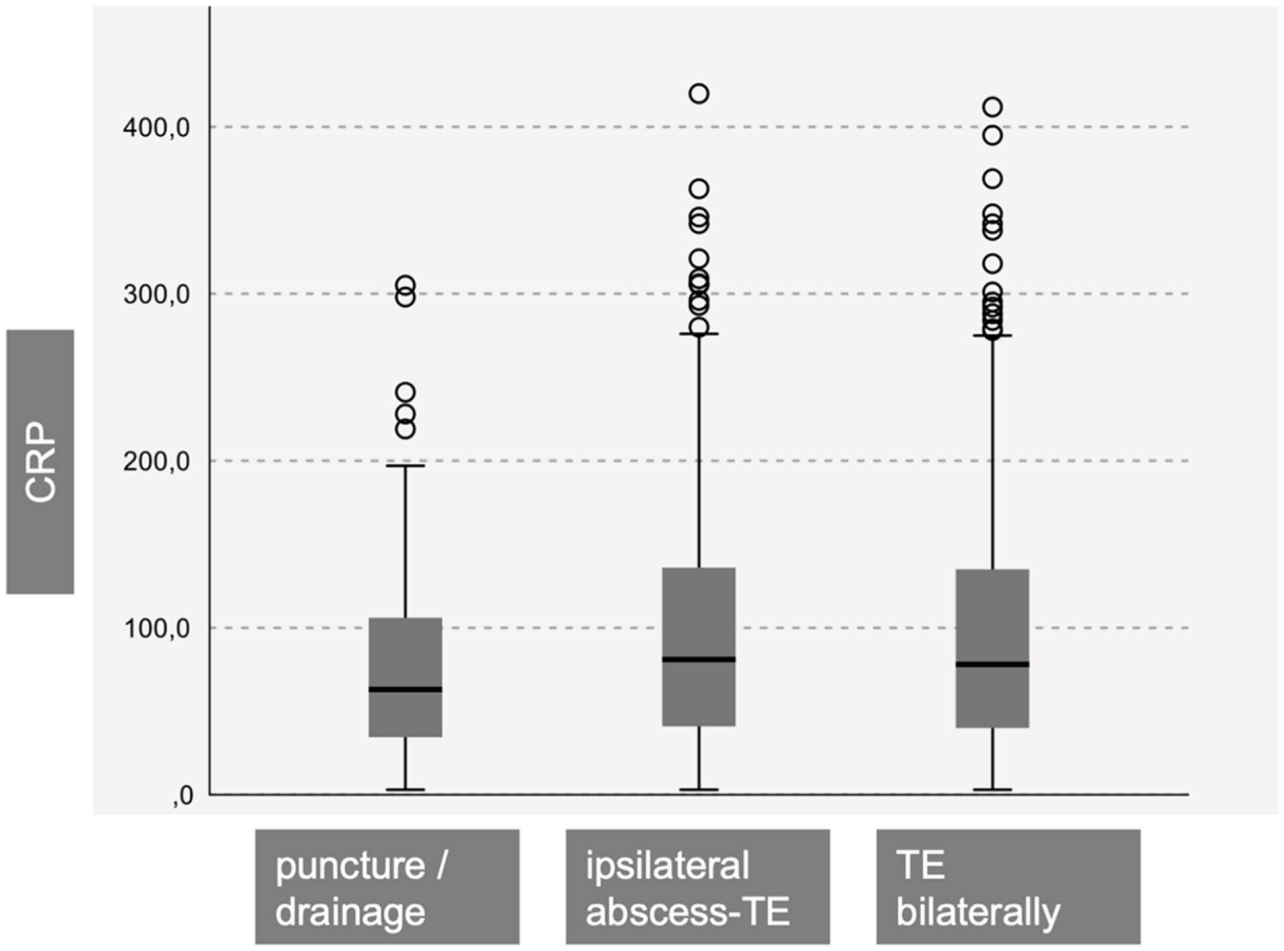
Patients with a successful needle aspiration (puncture) or incision and drainage did not show significant different CRP levels compared to ipsilateral abscess- and bilateral TE patients (CRP = C-reactive protein, TE = tonsillectomy).

As shown in Table 2, 180 patients were successfully treated with NA or ID. Among them, 42 underwent needle aspiration (NA-group), and 138 patients had additional incision under local anesthesia (ID-group). There were 291 patients in the UTE-group and 350 patients in the BTE-group. When comparing these three cohort groups, a significant difference was found in their mean age. Patients in the BTE-group were significantly younger. Additionally, patients in the NA-group and ID-group had significantly reduced leukocyte counts on the initial day of their inpatient stay compared to UTE-group and BTE-group patients (Table 2 and Figure 2). No significant differences were found regarding the C-reactive protein (Figure 3).

**Table 2:** Treatment during the initial inpatient stay of the entire study group (*n* = 821) was divided into patients who underwent needle aspiration and drainage, as well as patients who underwent tonsillectomy for unilateral and bilateral abscesses (PTA = peritonsillar abscess, TE = tonsillectomy, CRP = C-reactive protein, NA-group = needle aspiration group, ID-group = incision and drainage group).

	NA-/ID-groups		Ipsilateral abscess-TE	Bilateral TE
Total (*n*)	180		291	350
	NA-group	ID-group		
	42	138		
Age at PTA	36.15		34.65	28.01
Leucocytes (mean)	12.4		14.35	13.7
CRP (mean)	63		81	78

Regarding the 180 patients treated with NA and ID, it is crucial to examine how many cases required abscess TE in the short term. Table 3 presents the treatment at the initial inpatient stay of the NA or ID study cohort (*n* = 180). More than 50% of the patients initially treated with NA underwent TE between day 1 and 3 after the first intervention. However, 71% of the patients who underwent incision were successfully treated without needing TE. In total, 37.7% of the NA and ID patients underwent TE during the same inpatient stay. Sixteen patients underwent contralateral elective TE following ipsilateral abscess TE. Only two patients underwent bilateral elective TE after successful drainage of a PTA.

**Table 3:** Treatment during the initial inpatient stay of the group that underwent puncture and drainage (*n* = 180).

Initial therapy	Course during initial inpatient stay	Number of patients (n)
Needle aspiration (*n* = 97)	Definitive treatment	42 (43.3%)
	Ipsilateral abscess-TE	48 (49.5%)
	Bilateral TE	7 (7.2%)
Incision and drainage (*n* = 192)	successful	138 (71.9%)
	Ipsilateral abscess-TE	12 (6.2%)
	Bilateral TE	42 (21.9%)

More than 50% of the patients initially treated with needle aspiration later underwent tonsillectomy between 1 and 3 days. However, over 71% of the patients treated with drainage were successfully treated and did not require tonsillectomy (PTA = peritonsillar abscess, TE = tonsillectomy).

**Figure 4:**
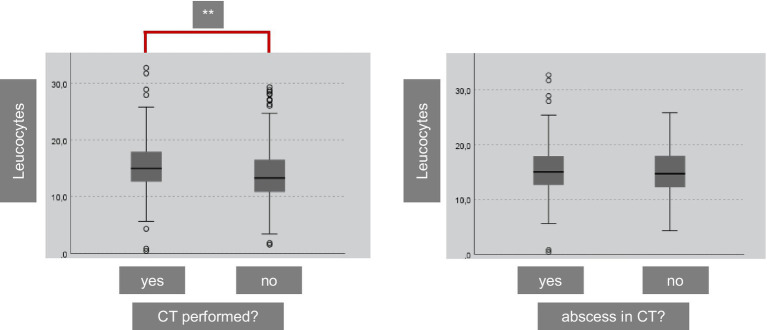
A CT was performed in 120 of the included 821 patients. We performed a CT significantly more often in patients with higher leucocytes (p < 0.001). Nevertheless, a clear abscess was only visible in 83.6% of the patients (CT = computed tomography).

PTA is a clinical diagnosis; however, CT scans were performed in 120 patients. This was significantly more frequent when leukocyte values were elevated at the first presentation (*p* < 0.001, Figure 4). Nonetheless, only 83.6% of the patients showed clear evidence of an abscess in the CT scan, despite clinical descriptions of an abscess. If no CT scan was performed, only 9 patients underwent NA or ID, and 101 patients underwent TE.

### Bleeding rates, inflammation, and complications

Within the UTE- and BTE-group, a total of 73 postoperative bleedings occurred. Out of these, 45 were observed after abscess TE, and 28 occurred after TE on the contralateral side of the PTA. Eight of these patients were anticoagulated, but none had a congenital coagulopathy. Therefore, the risk of secondary bleeding after TE was 11.4%. One patient continued to bleed after ID and required bipolar coagulation under local anesthesia. However, during the same inpatient stay, this patient was newly diagnosed with a factor VII deficiency.

Out of the 821 included patients, a total of 164 reported recurrent acute tonsillitis. The remaining 657 patients had no history of acute tonsillitis prior to PTA. However, none of the patients included here had 6 or more episodes of tonsillitis requiring antibiotics, which would be consistent with the recommendation for bilateral TE according to the in 2015 established and in 2019 updated German guideline (German guideline). All 164 patients who complained of recurrent acute tonsillitis underwent TE and were not treated with NA or ID.

Especially after bilateral TE, 105 patients complained of recurrent episodes of sore throat, which were coded and documented as pharyngitis by the ENT and/or family doctor. Among these patients, 24 reported annual sick leave due to these episodes of sore throat. These patients stated that they missed work for an average of 10.7 days per year, which was confirmed by their general practitioner. None of the patients in the ID- and NA-group reported recurrent acute tonsillitis, episodes of sore throat, or pharyngitis (compared to the BTE- and UTE-groups: *p* < 0.01).

### Long-term results of drainage

Overall, PTA recurred ipsilateral in only 5 patients during the follow-up period. Out of the 180 patients treated with NA or ID, two patients underwent elective TE on both sides. This resulted in a recurrence rate of 2.8% (5/178). Among these 5 patients, two initially had NA and three had ID. All patients relapsed between 3 months and 1.5 years. No second contralateral PTA was found in any of the patients during the observation period.

## Discussion

### Study cohort, comparison of treatment options, and diagnostic tools

The primary objective of this study was to investigate the long-term success rate of NA and/or ID in patients with PTA. This study boasts the largest original, single-center study cohort published to date, surpassing previous studies with smaller cohorts.

To date, the choice of the initial most appropriate surgical procedure for abscess drainage has not been clearly recommended (11). Overall, the goal must be safe abscess drainage (11). There is no mandatory in-house guideline for the treatment of peritonsillar abscesses. The decision as to which procedure was initially used therefore depended on various factors: Location of the abscess, patient history and compliance, and surgical skill.

Hahn et al. (12) included 584 patients in their retrospective analysis, reporting on 236 patients who underwent bilateral TE and 225 patients with unilateral abscess TE. They also mentioned that ID can be an effective treatment with the possibility of recurrence, as 71.4% of the patients treated with ID had successful outcomes without needing TE during the same hospital stay. Our study findings support these data, with over 71% of the patients in the ID-group successfully treated for PTA without requiring TE during a much longer follow-up. While Hahn et al. (12) solely relied on in-house electronic patient records, we combined subjective data from questionnaires with objective data from our in-house system and interviews with the patients’ general practitioners and ENT doctors. The 22% return rate for the patients’ questionnaires is considered satisfactory for a paper-based survey without any repeat reminder methods or potential lottery (13), especially considering the mobility of today’s society and the inclusion of patients up to the year 2007. Meta-analyses have shown that, among other things, short questionnaires are associated with higher response rates than longer ones (14). Nevertheless, the response rate of our study was below the average that can be achieved in patient surveys (14). The potential information gap was at least reduced by contacting the general practitioners.

**Figure 5:**
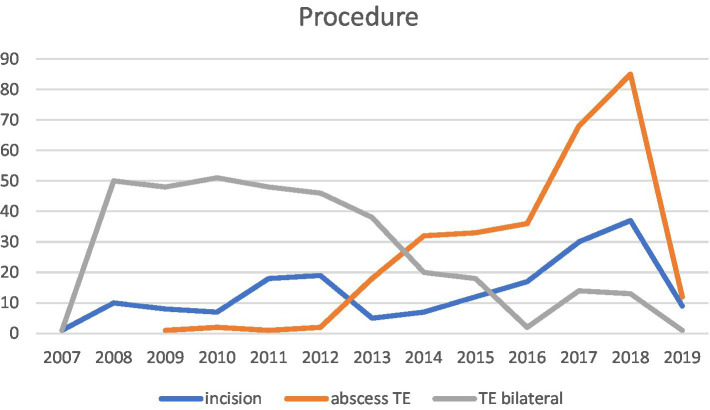
This illustration shows the time trend of performed procedures by year.

Moreover, Hahn et al. (12) noted a shift in PTA management in accordance with the German guideline in 2015, moving away from simultaneous bilateral TE in unilateral PTA. Our data also supports this shift, showing management moving away from bilateral TE in unilateral PTA starting from the year 2015 (see Figure 5).

Our data revealed that relying on laboratory parameters such as leukocyte count and C-reactive protein levels for the decision on whether TE is necessary or if ID are sufficient is not feasible, as there is significant overlap in the ranges for each cohort (Table 2, Figures 2, 3). Figure 2 shows that the NA−/ and ID- group shows significant diminished leucocyte counts in mean, but clinical relevance is not given due to the large overlap within the cohorts. Therefore, using initial leukocyte count as a definitive indicator for the success of NA, ID, or TE is not reliable. In 120 patients with suspected PTA complications, such as parapharyngeal abscess, a CT scan was performed. However, only 83.6% of the patients showed clear evidence of PTA in the CT scan, suggesting that escalation of therapy to unilateral or even bilateral TE based solely on the CT scan results should be thoroughly questioned. In fact, 16.4% of patients with suspected complications did not even have an obvious peritonsillar abscess. In our opinion, a CT scan is useful in suspected complications such as parapharyngeal abscess. However, in everyday clinical practice, it is not always possible to distinguish between, for example, an extensive peritonsillar abscess or a phlegmonous inflammation in the context of acute tonsillitis. Therefore, depending on the physician’s level of experience and the availability of computed tomography, unnecessary imaging may occur in some cases. However, indications for surgical intervention must always be made in the context of an appropriate clinic (e.g., trismus, unilateral odynophagia).

### Bleeding rates, inflammation, and complications

The overall postoperative hemorrhage rate following TE was 11.4% (73 out of 643 patients). No statistically significant difference was found between abscess TE and elective contralateral TE in the BTE-group. Hahn et al. (12) reported a postoperative hemorrhage rate of 11.5% (56 out of 489 patients). Previous studies described lower bleeding rates following TE and abscess TE ranging from 3.6 to 6%, but those studies had much smaller cohorts (15–19). Our study, with a total of 643 TE patients, demonstrates comparable results to Hahn et al. (12), which included 489 patients, supporting a realistic risk of postoperative hemorrhage of around 11 to 12%.

37.7% of the NA and ID patients underwent TE during the same inpatient stay. In the case of a recurrent abscess, the procedure depended on the estimated extent of the abscess, the possibility of re-incision/reopening of the drainage site, and the patient’s preference.

Among the 821 included patients, 657 reported no acute recurrent tonsillitis prior to PTA. This data was confirmed by their general practitioners and ENT doctors. None of the 164 patients with recurrent episodes of sore throat and/or recurrent acute tonsillitis demonstrated valid data of 6 or more acute tonsillitis cases requiring antibiotics, which would indicate the indication for bilateral TE according to the German guideline. None of the patients within the ID-group reported acute tonsillitis or episodes of sore throat. Burton et al. (20) described a reduction in sore throat episodes in adults due to TE in recurrent acute tonsillitis, but the quality of the data was rated as low, and the effect of TE was classified as “modest” (21). Koskenkorva et al. (17) recommended TE for individuals with 3 or more pharyngitis episodes and compared a TE group versus a waiting list. However, as in many studies, no precise differentiation was made between tonsillitis and pharyngitis.

In our study, within the BTE-group, 105 patients reported recurrent episodes of sore throat following TE, which were coded and documented as pharyngitis by the ENT and/or general practitioner. Among these patients, 24 reported annual sick leave due to these sore throat episodes, resulting in an average of 10.7 days of absence from work per year, confirmed by their general practitioner. Therefore, we conclude that unindicated bilateral TE can lead to increased sore throat episodes, resulting in work absences and a burden on our economy. Due to the partial overlap of symptoms, it is quite possible that some patients also had undiagnosed laryngopharyngeal reflux (22). Therefore, overinterpretation of a sore throat as a specific symptom of tonsillitis is possible. However, the data were based on information provided by the patients or general practitioners, which is why a clinically clean distinction between bacterial/viral inflammatory or reflux is not possible.

### Long-term results of incision and drainage

The results of our study demonstrate low recurrence rates of only 2.8% after ID (5 out of 178). None of the included patients experienced a contralateral PTA recurrence. In contrast, Hahn et al. (12) reported 6 patients with abscess recurrence following ID of a PTA within 2 months and up to 2 years, with only 8 out of 584 patients (1.4%) having a history of treated PTA before their retrospective analysis, and none of them showing a clearly documented contralateral PTA recurrence.

Whereas Herzon et al. (23) described successful rates for NA in 123 patients, Johnson et al. (24) reported in their meta-analysis NA with 10–15% recurrence rates. A French study of Mansour et al. (25) reported of 14 recurrences following NA and 4% recurrence rate following ID in their study cohort. Khan et al. (26) reported of 60 patients with a successful PTA drainage. Nevertheless, all recommendations are based on retrospective study data and small study cohorts (23, 27, 28).

The German guideline suggests that ID or abscess TE are equivalent options for treating a PTA and are indicated depending on patient compliance and other risk factors. NA or ID are preferred for patients with an increased risk of surgery, anesthesia, comorbidities, or coagulation disorders, while abscess TE is recommended when other options fail. Bilateral TE should only be considered for cases of bilateral PTA or recurrent acute tonsillitis on the contralateral side with six or more episodes in the last 12 months. Other guidelines from various countries offer different recommendations, but none explicitly reject ID as a treatment option. The French guideline (29) describes that TE is indicated in cases of chronic tonsillitis and PTA, but also reports of a low level of evidence. Italian recommendations (30) consider TE earlier in cases with acute tonsillitis and / or PTA. American guidelines, published in 2011 recommend TE strictly regarding the Paradise criteria, but name the PTA as the only exception. Nevertheless, neither the American (31), nor the French, nor the Scottish (32–34) guideline recommended, discussed or refused the treatment option via ID.

Our study adds valuable insights to the existing guidelines. We demonstrated that ID is successful in 71% of cases and should be considered not only for patients with comorbidities but for all patients with a singular PTA without signs of complications such as a parapharyngeal abscess and without a history of recurrent acute tonsillitis.

### Limitations

Although this study represents the largest original cohort published so far, it is a single-center retrospective analysis. Documentation was sometimes inadequate and inconsistent, relying on surgical reports and documentation from colleagues. Additionally, the retrospective questioning of patients after 12 years may introduce biases and the below-average return rate can cause a selection bias. However, being a single-center study allows for uniform documentation and a consistent approach to therapy. Furthermore, we included adults and children and a selection bias due to age and compliance cannot be excluded.

## Conclusion

During the consultation with the patients, ID should be offered as a less complicated alternative compared to abscess TE for PTA treatment, as the risk of postoperative bleeding after TE is high (11%), leading to longer absence from work and a longer hospital stay. Moreover, patients who undergo bilateral TE report higher rates of incapacity to work due to sore throat caused by pharyngitis. None of the patients fulfilled the clear indication for bilateral TE due to recurrent acute tonsillitis. The PTA recurrence rate after ID was very low ipsilaterally (2.8%) and zero contralaterally.

## Data availability statement

The raw data supporting the conclusions of this article will be made available by the authors, without undue reservation.

## Ethics statement

The studies involving humans were approved by Ethik-Kommission der Albert-Ludwigs-Universität Freiburg Engelberger Straße 2,179,106 Freiburg. The studies were conducted in accordance with the local legislation and institutional requirements. Written informed consent for participation in this study was provided by the participants’ legal guardians/next of kin. Written informed consent was obtained from the individual(s), and minor(s)’ legal guardian/next of kin, for the publication of any potentially identifiable images or data included in this article.

## Author contributions

MK: Conceptualization, Data curation, Formal analysis, Investigation, Methodology, Writing – original draft, Writing – review & editing. MM: Data curation, Formal analysis, Project administration, Writing – review & editing. VB: Data curation, Writing – review & editing. NM: Writing – review & editing. AK: Conceptualization, Resources, Writing – review & editing. CB: Conceptualization, Project administration, Writing – original draft, Writing – review & editing.
